# Case Report: Controversies in managing pulmonary enteric adenocarcinoma: reflections from an early-stage case

**DOI:** 10.3389/fonc.2025.1603084

**Published:** 2025-06-17

**Authors:** Yuchen Bao, Dongdong Liu, Yanzhe Wang, Zhigang Wu, Haitao Wang

**Affiliations:** ^1^ Hangzhou Normal University, Hangzhou, Zhejiang, China; ^2^ Cancer Center, Department of Thoracic Surgery, Zhejiang Provincial People’s Hospital, People’s Hospital of Hangzhou Medical College, Hangzhou, Zhejiang, China; ^3^ Zhejiang Chinese Medical University, Hangzhou, Zhejiang, China

**Keywords:** pulmonary enteric adenocarcinoma (PEAC), PET-CT, multidisciplinary diagnosis, surgical resection, case report

## Abstract

Pulmonary enteric adenocarcinoma (PEAC) is a rare non-small cell lung cancer subtype characterized by predominant intestinal differentiation (≥50%) and histological resemblance to colorectal adenocarcinoma. We report a 70-year-old male ex-smoker with an incidentally detected 18×11 mm spiculated lung nodule on chest CT, which subsequently demonstrated intense FDG uptake (SUVmax 14.0) on PET-CT. Histopathological evaluation confirmed PEAC. Immunohistochemistry revealed HER2 overexpression (3+) and intestinal differentiation markers (CK7+, CK20+, CDX2+, Villin+), while molecular testing showed wild-type ERBB2 and no actionable mutations. The patient underwent successful R0 resection with no recurrence at 8-month follow-up. This case underscores the critical importance of a multimodal diagnostic approach integrating immunohistochemical markers (notably CK7’s superior specificity), PET-CT imaging, and endoscopic evaluation to reliably differentiate PEAC from metastatic gastrointestinal malignancies. Furthermore, the patient’s favorable outcome following R0 resection without adjuvant therapy reinforces surgical intervention as the cornerstone of treatment for localized PEAC, particularly in early-stage disease. Advanced cases require early multidisciplinary collaboration to develop individualized treatment.

## Background

Pulmonary enteric adenocarcinoma (PEAC) is a rare NSCLC subtype defined by predominant (≥50%) intestinal-type differentiation and histological similarity to colorectal adenocarcinoma in primary lung tumors (1). PEAC is relatively rare in clinical practice, with an overall prevalence of approximately 0.6%-0.68% in primary pulmonary adenocarcinoma (2, 3). Literature reports that this disease is more common in middle-aged and elderly people (2). Currently, there are fewer than 500 cases of PEAC described in English scientific literature (4), primarily in the form of case reports or small case series, and literature related to the treatment of PEAC is even scarcer.

## Case presentation

**Figure 1:**
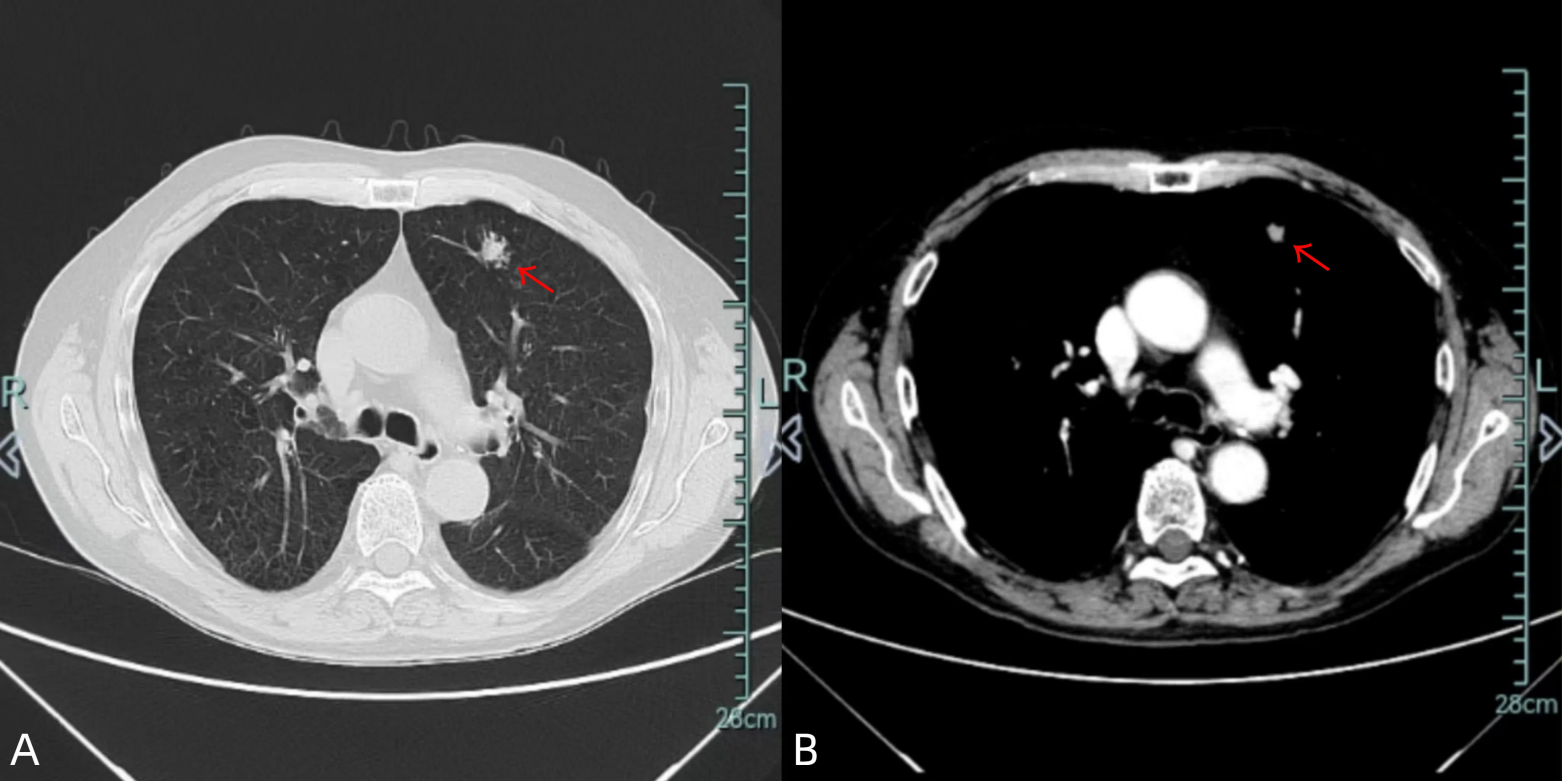
Chest CT images [lung window **(A)**/mediastinal window **(B)**] demonstrate an 18×11 mm spiculated nodule in the left upper lobe (red arrow).

A 70-year-old male ex-smoker (30 pack-years) presented with an incidentally detected 18×11 mm spiculated nodule in the anterior segment of the left upper lobe on chest CT (**Figure 1**). CT-guided biopsy confirmed adenocarcinoma, prompting further evaluation to exclude metastatic disease. Comprehensive endoscopic evaluation including esophagogastroduodenoscopy and colonoscopy showed no evidence of malignancy or other clinically relevant pathology.

**Figure 2:**
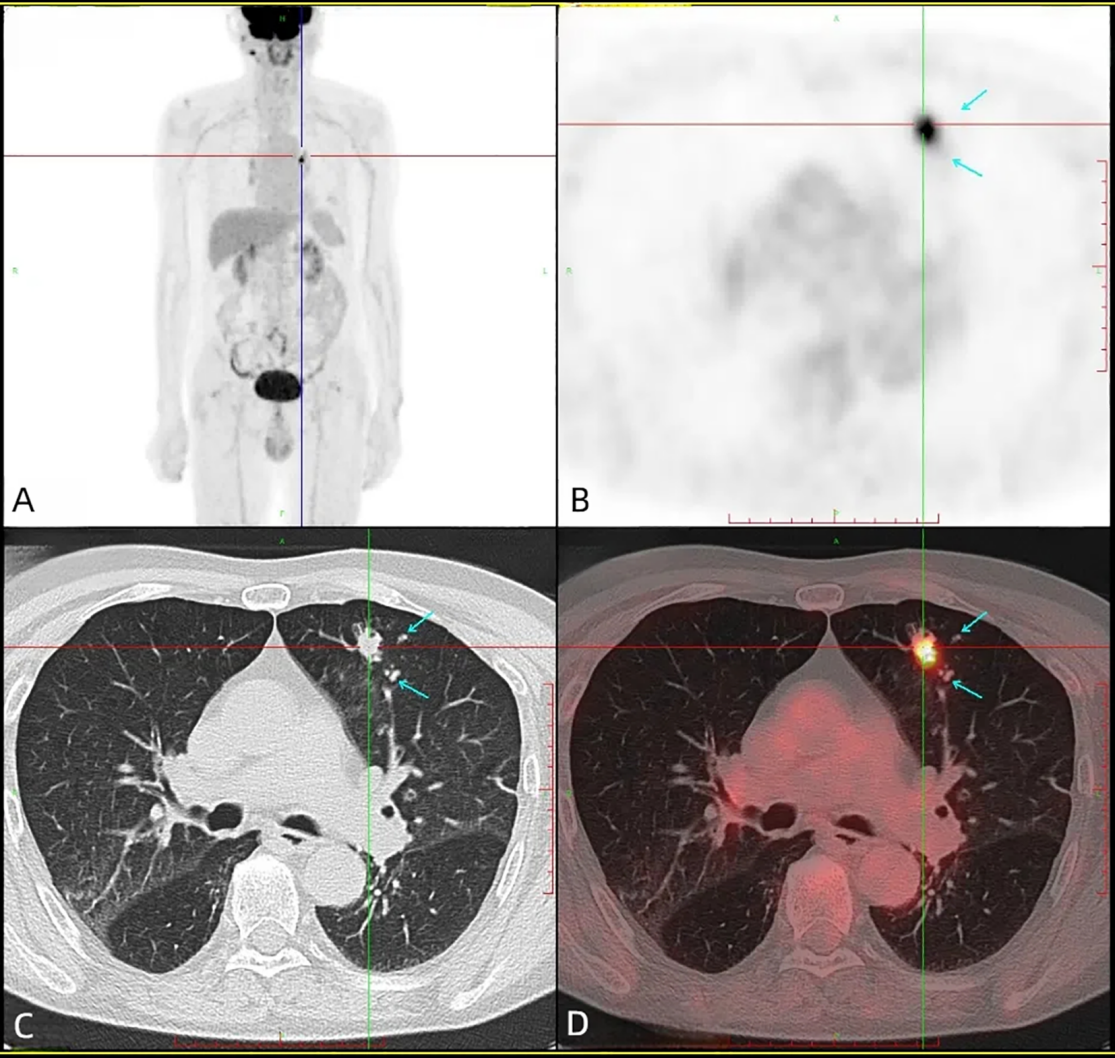
Head + torso 18F-FDG PET/CT revealed a heterogeneous FDG uptake in the left upper lobe (SUVmax = 14.0), with no evidence of hypermetabolic lesions in the gastrointestinal tract. **(A)** PET maximum intensity projection (MIP) image, **(B)** axial PET image, **(C)** axial CT image, **(D)** axial fusion image.

PET-CT demonstrated intense FDG uptake (SUVmax 14.0) in the dominant lesion without metabolically active satellite nodules (**Figure 2**). Serum tumor markers (CEA, CA19-9, CYFRA21-1, NSE) were within normal ranges.

**Figure 3:**
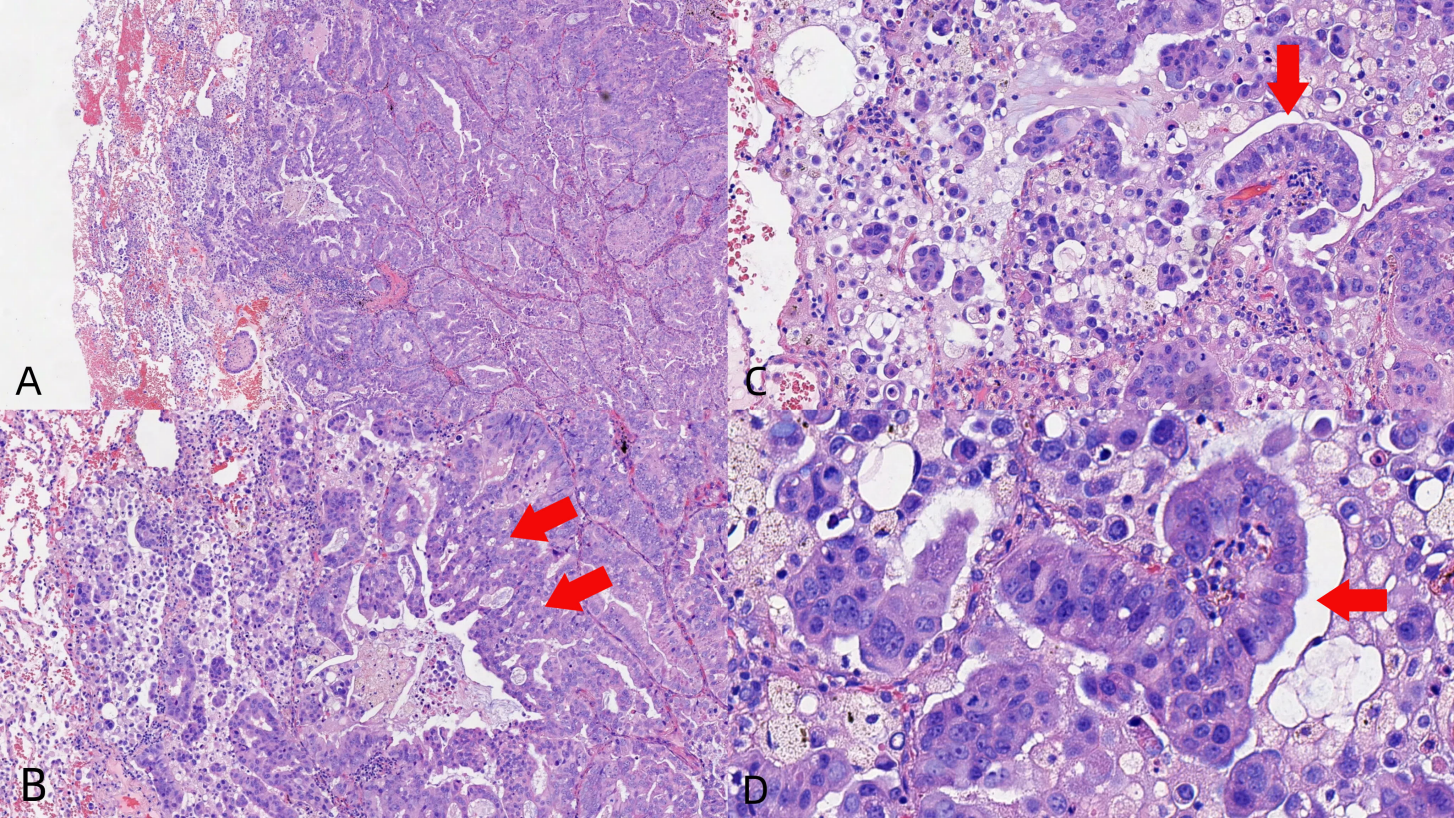
Core biopsies showing the tumor with glandular architecture [H&E stain, power of magnification ×100 **(A)**, ×200 **(B, C)**, and ×400 **(D)**].

Thoracoscopic wedge resection of the left upper lobe revealed a 1.5×1.2×1 cm invasive adenocarcinoma with negative surgical margins. Postoperative staging was pT1bNxMx (AJCC 8th edition). Histopathological evaluation demonstrated intestinal differentiation features (**Figure 3**) with the following characteristics: neural invasion (-), lymphovascular invasion (+), no visceral pleural invasion (PL0), and tumor spread through air spaces (STAS). Immunohistochemical profile: HER-2(3+), CK7(+), TTF-1(-), NapsinA(-), P40(-), CK5/6(-), Ki67(+,60%), ALK(D5F3)(-), ALK-NC(-), ALK-PC(+), P53(+) (mutant type), CDX2(+), CK20(+), Villin(focal+), Muc-2(-), SATB2(-). PD-L1 tumor proportion score was <1%.

A 10-gene ARMS-PCR panel (EGFR, ALK, ROS1, RET, KRAS, NRAS, BRAF, ERBB2/HER2, PIK3CA, MET) detected no clinically actionable mutations or fusions at a 1% sensitivity threshold. It is noteworthy that HER2 protein overexpression (3+) contradicted the wild-type ERBB2 gene status.

The patient received postoperative surveillance without adjuvant therapy, and no evidence of recurrence was observed at the 8-month follow-up.

## Discussion and conclusion

Pulmonary enteric adenocarcinoma (PEAC), also known as the intestinal variant of lung adenocarcinoma, is a very rare subtype of NSCLC. It was first described by Tsao and Fraser in 1991 (5) and was officially classified as a histological type of lung adenocarcinoma in the 2015 World Health Organization histological classification of lung tumors. According to the WHO criteria (1), the diagnosis of PEAC requires: 1.Primary pulmonary origin; 2. ≥50% tumor composition showing intestinal-type differentiation; 3.Morphological overlap with colorectal adenocarcinoma.

Studies have shown that the incidence of PEAC is higher in middle-aged and elderly patients and more common in males (2), though some scholars believe the incidence is similar between sexes (6). Due to the small number of reported cases, sex-specific incidence rates remain controversial. Smokers are more likely to develop PEAC than non-smokers (6), and our patient also had a long-term smoking history, consistent with research findings.

Cough is the most common initial symptom of PEAC, followed by back pain, chest pain, and hemoptysis (2, 7–10). Other symptoms include chest tightness, dyspnea, fever, night sweats, throat discomfort, headache, fatigue, and cervical lymphadenopathy (11). Gastrointestinal symptoms such as nausea, vomiting, abdominal pain, diarrhea, constipation, or bloody stools were not observed (12); thus, PEAC patients are typically first diagnosed in respiratory departments. However, our patient was incidentally found to have a nodule during a physical examination at an external hospital and was asymptomatic upon admission to our institution, which may relate to the tumor’s peripheral location, small diameter, and lack of pleural invasion.

Previous studies have reported varying degrees of elevated serum tumor markers in PEAC patients, particularly CEA and CA19-9. In contrast, CYFRA21–1 and NSE, which are relatively sensitive and specific for lung cancer, showed no significant changes (9, 13). According to statistics from Li H et al., CEA levels were elevated in 68.2% (45/66) of patients, and CA19–9 levels were elevated in 48.4% (15/31) of patients. CYFRA21–1 and NSE were rarely positive, at approximately 10% (2/20) and 0% (0/19), respectively (9). Preoperative tumor marker testing in this patient revealed no abnormally elevated indicators, possibly related to the early stage of the disease.

Observations in existing cases indicate that CEA levels significantly decrease after chemotherapy in advanced PEAC patients with distant metastasis (12, 14), suggesting this marker may help monitor clinical progression. Postoperative re-examination at 3 months showed the patient’s CEA decreased from 4.7 ng/mL preoperatively to 4.4 ng/mL, both within normal ranges. Thus, CEA may not be an ideal marker for monitoring early-stage PEAC.

Since the lung is a common metastatic site for colorectal cancer, clinicians must rule out colorectal-origin malignancies when diagnosing PEAC. Studies show that immunohistochemical markers such as Villin, CK7, CK20, and CDX2 have significantly higher positivity rates in PEAC than in metastatic colorectal cancer (MCC) (15). CK7 is particularly critical for differentiating PEAC from MCC, as it exhibits better specificity than CK20 and CDX2 (6, 16). However, due to morphological, immunohistochemical, and even genetic similarities between PEAC and colorectal adenocarcinoma (15), distinguishing them based solely on histopathology is challenging (17–19). A definitive PEAC diagnosis requires pathological examination of pulmonary lesions combined with systemic multi-organ evaluations to exclude metastatic gastrointestinal tumors. Feng C et al. emphasized that origin cannot be determined by morphological features alone; accurate diagnosis requires comprehensive clinical examinations, immunohistochemical staining, and genetic testing (15). Stojsic J et al. consider colonoscopy one of the most valuable methods for diagnosing PEAC (17). The final diagnosis should integrate the patient’s medical history and clinical evaluations, including CT, fiberoptic endoscopy, and PET-CT, to exclude primary colorectal cancer. In this case, the patient underwent lung biopsy at an external hospital, with pathology results suggesting “adenocarcinoma, metastatic origin to be excluded.” Although initially suspected to be a metastatic lesion, gastrointestinal endoscopy showed no evidence of malignancy or other clinically significant pathology. Ultimately, PET-CT imaging ruled out metastatic disease from other primary sites and confirmed the nodule as a “primary lung malignancy. “After confirming the primary tumor, surgical resection was performed. Based on this patient’s experience, we emphasize the diagnostic value of PET-CT for early-stage PEAC and recommend its use for definitive diagnosis in all PEAC cases.

PEAC is a rare subtype of primary pulmonary adenocarcinoma. Internationally, no specific treatment guidelines exist for PEAC, and most clinicians follow NSCLC protocols. Surgical intervention is the preferred treatment for early-stage patients, supported by data from several studies (7, 9). Advanced patients often receive surgery combined with chemotherapy, typically platinum-paclitaxel regimens. Given the patient’s poor pulmonary function (with severe ventilatory dysfunction and markedly reduced diffusing capacity of the lungs for carbon monoxide [DLCO]) and an ECOG performance status of 2, we opted for thoracoscopic wedge resection instead of anatomical resection and omitted lymph node dissection. Intraoperatively, no enlarged lymph nodes were identified under video-assisted thoracoscopic surgery (VATS), further supporting the rationale for forgoing lymphadenectomy. The postoperative pathology of this patient revealed the presence of lymphovascular invasion (LVI+) and spread through air spaces (STAS). Studies have shown that LVI-positive patients have higher risks of recurrence and mortality compared to LVI-negative patients (20, 21). Furthermore, the 2020 NCCN Guidelines recommended adjuvant chemotherapy for high-risk patients with LVI (22). The 2023 NCCN Guidelines, for the first time, explicitly listed STAS as a high-risk factor in the pathology section and suggested considering adjuvant therapy for stage IB or higher patients with STAS (22). Given that this patient was considered stage IA2 and had poor baseline conditions, adjuvant therapy was not administered. As of the 8-month postoperative follow-up, there has been no evidence of recurrence. However, it should be noted that the follow-up data in this case report is relatively short, which represents a limitation of this study. Although targeted therapy has been proposed as an important approach in recent years, few patients have received such treatments. Fassi E et al. identified high-frequency target gene mutations in PEAC and recommended gene panel analysis for all cases (7). Postoperative molecular profiling in this patient revealed no detectable gene fusions or mutations. Notably, we identified a discordance between HER2 protein overexpression (IHC 3+) and negative molecular findings (wild-type ERBB2 status), which has not been previously documented in published PEAC case reports. This discrepancy has been frequently documented in breast cancer (23, 24). As the existing literature lacks direct evidence explaining this phenomenon in PEAC, the precise underlying mechanisms remain unclear. Additionally, external factors such as limitations in detection methods cannot be disregarded. For instance, the HER2 status may vary across different regions of the lesion, leading to discrepancies between the biopsy sample and the overall tumor characteristics.For early-stage PEAC patients, we recommend prompt surgical intervention to excise lesions, confirm diagnosis, and guide postoperative management. Adjuvant chemotherapy is generally unnecessary for R0-resected early-stage patients. This patient has undergone regular follow-up at our institution with no recurrence. For advanced patients, early multidisciplinary discussions are essential to formulate personalized treatment plans.

In conclusion, PEAC is a rare disease. Preoperative diagnosis should not rely solely on immunohistochemistry but requires comprehensive evaluation of patient history and clinical examinations to exclude gastrointestinal malignancies. Complete surgical resection is the preferred treatment for early-stage patients, while advanced cases necessitate early multidisciplinary collaboration to develop individualized therapies.

## Data Availability

The original contributions presented in the study are included in the article/supplementary material. Further inquiries can be directed to the corresponding author.
